# Association between *HLA* alleles and beta-lactam antibiotics-related severe cutaneous adverse reactions

**DOI:** 10.3389/fphar.2023.1248386

**Published:** 2023-09-19

**Authors:** Pansakon Wattanachai, Warayuwadee Amornpinyo, Parinya Konyoung, Danklai Purimart, Usanee Khunarkornsiri, Oranuch Pattanacheewapull, Wichittra Tassaneeyakul, Nontaya Nakkam

**Affiliations:** ^1^ Department of Pharmacology, Faculty of Medicine, Khon Kaen University, Khon Kaen, Thailand; ^2^ Division of Dermatology, Department of Internal Medicine, Khon Kaen Hospital, Khon Kaen, Thailand; ^3^ Pharmacy Unit, Udon Thani Hospital, Udon Thani, Thailand; ^4^ Pharmacy Department, Khon Kaen Hospital, Khon Kaen, Thailand

**Keywords:** beta-lactam antibiotics, severe cutaneous adverse reactions, Stevens–Johnson syndrome/toxic epidermal necrolysis, acute generalized exanthematous pustulosis, drug reactions with eosinophilia and systemic symptoms, Human Leukocyte Antigen

## Abstract

**Introduction:** Beta-lactam antibiotics are one of the most common causes of antibiotics-related severe cutaneous adverse reactions (SCARs) including Stevens–Johnson syndrome (SJS), toxic epidermal necrolysis (TEN), drug reactions with eosinophilia and systemic symptoms (DRESS), and acute generalized exanthematous pustulosis (AGEP). Recent evidence demonstrated that the human leukocyte antigen (*HLA*) polymorphisms play important roles in the development of drug-related SCARs. This study aimed to extensively characterize the associations between *HLA* genetic polymorphisms and several phenotypes of SCARs related to beta-lactam antibiotics.

**Methods:** Thirty-one Thai patients with beta-lactam antibiotics-related SCARs were enrolled in the study. A total of 183 unrelated native Thai subjects without any evidence of drug allergy were recruited as the control group. Genotyping of *HLA* class I and class II alleles was performed.

**Results:** Six *HLA* alleles including HLA-A*01:01, HLA-B*50:01, HLA-C*06:02, HLA-DRB1*15:01, HLA-DQA1*03:01, and HLA-DQB1*03:02, were significantly associated with beta-lactam antibiotics-related SCARs. The highest risk of SCARs was observed in patients with the HLA-B*50:01 allele (OR = 12.6, 95% CI = 1.1–142.9, *p* = 0.042), followed by the HLA-DQB1*03:02 allele (OR = 5.8, 95% CI = 1.5–22.0, *p* = 0.012) and the HLA-C*06:02 allele (OR = 5.7, 95% CI = 1.6–19.9, *p* = 0.011). According to the phenotypes of SCARs related to beta-lactam antibiotics, the higher risk of SJS/TEN was observed in patients with HLA-A*03:02, HLA-B*46:02 (OR = 17.5, 95% CI = 1.5–201.6, *p* = 0.033), HLA-A*02:06, HLA-B*57:01 (OR = 9.5, 95% CI = 1.3–71.5, *p* = 0.028), HLA-DQB1*03:02 (OR = 7.5, 95% CI = 1.8–30.9, *p* = 0.008), or HLA-C*06:02 (OR = 4.9, 95% CI = 1.1–21.4, *p* = 0.008). While eight *HLA* alleles including HLA-A*02:05, HLA-A*02:11, HLA-B*37:01, HLA-B*38:01, HLA-B*50:01, HLA-C*06:02, HLA-C*03:09, and HLA-DRB1*15:01 were associated with AGEP, the highest risk of AGEP was observed in patients with the HLA-B*50:01 allele (OR = 60.7, 95% CI = 4.8–765.00, *p* = 0.005). Among the four *HLA* alleles associated with DRESS including HLA-C*04:06, HLA-DRB1*04:05, HLA-DRB1*11:01, and HLA-DQB1*04:01, the HLA-C*04:06 allele had the highest risk of beta-lactam antibiotics-related DRESS (OR = 60.0, 95% CI = 3.0–1202.1, *p* = 0.043). However, these associations did not achieve statistical significance after Bonferroni’s correction. Apart from the *HLA* risk alleles, the HLA-A*02:07 allele appeared to be a protective factor against beta-lactam antibiotic-related SCARs (OR = 0.1, 95% CI = 0.0–0.5, *p* = 3.7 × 10^−4^, Pc = 0.012).

**Conclusion:** This study demonstrated the candidate *HLA* alleles that are significantly associated with several phenotypes of beta-lactam antibiotics-related SCARs. However, whether the *HLA* alleles observed in this study can be used as valid genetic markers for SCARs related to beta-lactam antibiotics needs to be further explored in other ethnicities and larger cohort studies.

## 1 Introduction

Beta-lactam antibiotics are commonly prescribed medications with various clinical indications. However, they have been reported as the most common cause of antibiotic-induced cutaneous adverse drug reactions ranging in severity from mild, self-limited cutaneous eruptions such as urticaria and maculopapular exanthema (MPE) to life-threatening severe cutaneous adverse reactions (SCARs) including Stevens–Johnson syndrome (SJS), toxic epidermal necrolysis (TEN), drug reaction with eosinophilia and systemic symptoms (DRESS), and acute generalized exanthematous pustulosis (AGEP) (Blumenthal et al., 2019). SJS/TEN, DRESS, and AGEP vary in mortality, immunopathogenesis, and clinical characteristics. For SJS/TEN cases, cytotoxic CD8^+^ T cells accumulate in blisters and release granulysin, perforin, and granzyme B to kill keratinocytes. In the case of DRESS, CD4^+^ and CD8^+^ T cells, plasma dendritic cells (DCs), and monocytes are enriched in the dermis, and the release of TNF-α and IFN-γ was found. Moreover, interleukin 5 (IL-5) is produced from Th2 cells and group 2 innate lymphoid cells (ILC2s) in which IL-5 can induce the activation and migration of eosinophils that drive inflammation in DRESS. For AGEP, CD4^+^ T cells secrete IL-4, IL-5, IL-13, IFN-γ, TNF-α, IL-8, IL-17, and IL-22 (Th17). IL-8 drives neutrophil and the recruitment of T cells to the epidermis to form sterile pustules (Karnes et al., 2019; Gibson et al., 2023). These SCARs are delayed type IV hypersensitivity reactions or T-cell-mediated immune response, with a high mortality rate of less than 1% in AGEP, less than 10% in DRESS, and 15–50% in SJS/TEN. Moreover, patients who recover from SCAR episodes may be left with sequelae or long-lasting disabilities such as blindness (Roujeau, 2005; Chung et al., 2016; Gibson et al., 2023).

Recent evidence revealed that genetic polymorphisms of human leukocyte antigen (*HLA*) genes may play important roles in the development of drug-induced life-threatening SCARs. Some *HLA* alleles have been demonstrated to be strongly associated with drug-induced SCARs and have been proposed as valid genetic markers for the prediction of drug-induced SCARs such as HLA-B*15:02 for carbamazepine-induced SJS/TEN (Chung et al., 2004; Hung et al., 2006; Tassaneeyakul et al., 2010; Nakkam et al., 2022a), HLA-B*58:01 for allopurinol-induced SCARs (Hung et al., 2005; Tassaneeyakul et al., 2009; Saksit et al., 2017b), or HLA-B*57:01 for abacavir hypersensitivity (Mallal et al., 2002; Mallal et al., 2008). According to these strong associations, the regulatory agencies in several countries as well as the Clinical Pharmacogenetics Implementation Consortium (CPIC) suggest physicians to perform *HLA* screening tests in individual patients before the initiation of some drugs to identify patients who are at a higher risk of drug-induced SCARs (Saito et al., 2016; Phillips et al., 2018).

A recent study of 1,078 SJS/TEN Asian patients, including Thai patients, who have been admitted to hospitals during 1998–2017, from registration databases revealed that beta-lactam antibiotics, especially aminopenicillins and cephalosporins, are the most common cause of antibiotics-induced SJS/TEN after sulfamethoxazole (Wang et al., 2019). The associations between genetic markers and beta-lactam antibiotics-induced adverse drug reactions have been reported, in which the *HLA-DRB* gene appeared to be associated with IgE-mediated-type hypersensitivity to penicillin in a Chinese population (Yang et al., 2006). Consistent with the findings in a Chinese population, HLA-DRB1*10:01 alleles were associated with penicillin-induced immediate reactions in a European population (Nicoletti et al., 2021). Apart from the immediate reaction, the study in an Italian population demonstrated that the HLA-DRB3*02:02 allele was associated with penicillin-induced delayed hypersensitivity, especially with MPE (Romano et al., 2022). Moreover, HLA-B*55:01 was related to self-reported penicillin delayed reactions in a genome-wide association study in a European population, and it should be noted that the most common phenotype of skin reactions found in this study was delayed-type rash (Krebs et al., 2020).

In addition, HLA-B*48:01 was strongly associated with immediate reactions from beta-lactam hypersensitivity in Thai children, while HLA-C*04:06, HLA-C*08:01, and HLA-DRB1*04:06 were associated with beta-lactam antibiotic-induced delayed-type hypersensitivity reactions (Singvijarn et al., 2019). It should be noted that only two patients with DRESS and SJS were recruited in Singvijarn et al. (2019). Apart from co-trimoxazole, the data on associations between SCARs and *HLA* polymorphisms in other antibiotics are still limited. The present study aimed to extensively characterize the associations between *HLA* genetic polymorphisms, including *HLA* class I and class II, and several phenotypes of SCARs related to beta-lactam antibiotics in a Thai population.

## 2 Materials and methods

### 2.1 Study population

Patients with beta-lactam antibiotics-related SCARs, who were admitted to a local hospital in Thailand from 2009 to 2022, were recruited. Patients hospitalized between 2009 and 2019 were identified retrospectively by reviewing their medical records, whereas patients who were hospitalized between 2020 and 2022 were prospectively enrolled in the study. The diagnosis of SCARs was made primarily by an internist or a dermatologist in each hospital and subsequently confirmed by a dermatologist in the investigator team. The criteria for SJS, TEN, and DRESS were classified as previously described (Nakkam et al., 2022a; Nakkam et al., 2022b). While AGEP was defined as numerous non-follicular pustules arising on a widespread edematous erythema, fever, and elevated blood neutrophils, histopathology typically showed spongiform subcorneal and/or intraepidermal pustules, a marked edema of the papillary dermis, and eventually vasculitis, eosinophilia, and/or focal necrosis of keratinocytes (Sidoroff et al., 2001).

The causative drugs were identified by a corresponding Naranjo adverse drug reaction (ADR) score of ≥5 (probable ADR). Moreover, for SJS and TEN, the drug causality for each patient was evaluated based on the algorithm for the assessment of drug causality (ALDEN score ≥4; probable SJS/TEN) (Sassolas et al., 2010). The scoring system for classifying DRESS cases was based on RegiSCAR (Kardaun et al., 2007), and the scoring system for classifying AGEP cases was based on EuroSCAR (Sidoroff et al., 2001). All patients with probable RegiSCAR or EuroSCAR were enrolled as cases.

Thai subjects who had been using several medications including beta-lactam antibiotics without any evidence of drug allergy were recruited as a control group. Written informed consent was obtained from each participant. This study protocol was approved by the Ethics Committee for Human Research, Khon Kaen University, Thailand (HE510837).

### 2.2 Genomic DNA preparation

Peripheral blood samples were collected into EDTA-coated tubes. Buffy coats were separated by centrifugation at 3,500 RPM, 4°C, for 15 min. Genomic DNA was then isolated from leukocytes using QIAamp DNA Blood mini kits (QIAGEN GmbH, Hilden, Germany).

### 2.3 *HLA* genotyping

Genotyping of *HLA* class I, including *HLA-A*, *HLA-B*, and *HLA-C*, and *HLA* class II, including *HLA-DRB1*, *HLA-DQA1*, and *HLA-DQB1*, was determined using the LIFECODES *HLA* Typing Kits (Immucor GTI Diagnostics, Waukesha, Wisconsin, United States), which are based on the reverse sequence-specific oligonucleotide (SSO) probe method coupled with xMAP Technology designed to be used with the Luminex^®^ System, as previously described (Nakkam et al., 2022a; Nakkam et al., 2022b). The *HLA* alleles were analyzed using LIFECODES MATCH IT DNA Software version 1.3 based on Allele Database version 3.41. According to the previous studies (Wang et al., 2021; Nakkam et al., 2022a), it was reported that the results of two-field or four-digit resolution of *HLA* genotypes from LIFECODES HLA typing were concordant with the SeCore HLA sequence-based typing (SBT) method.

### 2.4 Data statistical analysis

The demographic data and clinical characteristics of SCAR cases were carried out using descriptive statistics such as percentage, median, mean, and standard deviation. The statistical analysis among SCAR phenotypes for the clinical characteristics was carried out using two-tailed Student’s *t*-test and Fisher’s exact test.

The allele frequencies and genotype frequencies of the *HLA* class I and II alleles were determined by direct counting. The strength of associations was estimated by calculating the odds ratios (ORs) and 95% confidence intervals (CIs) using SPSS statistical software, version 28.0 for macOS (IBM, Armonk, New York, United States). ORs were determined using Haldane’s modification, which adds 0.5 to all cells to accommodate possible zero counts. The corrected *p*-values (Pc) for the multiple comparisons of *HLA* (34 for *HLA-A*, 53 for *HLA-B*, 36 for *HLA-C*, 29 for *HLA-DRB1*, 10 for *HLA-DQA1*, and 15 for *HLA-DQB1*) were calculated using Bonferroni’s correction. All *p*-values were empirical, two-tailed values, and a value less than 0.05 was considered statistically significant. The estimated linkage disequilibrium coefficients (D’) and coefficient of correlation (*r* ^2^) among identified *HLA* alleles were calculated using the PLINK (V1.07) program (Ardlie et al., 2002; Saksit et al., 2017a).

## 3 Results

### 3.1 Characteristics of the study population

Thirty-one beta-lactam antibiotics-related SCARs patients who met the inclusion criteria were enrolled in this study. Among these, 21 patients (67.7%) were diagnosed with SJS/TEN (13 with SJS, one with overlap SJS/TEN, and seven with TEN), eight patients (25.8%) were diagnosed with AGEP, and two patients (6.5%) were diagnosed with DRESS.

The beta-lactam antibiotics-related SCARs patients comprised of nine men and 22 women with an average age of 52.1 ± 22.1 years (47.3 ± 24.5 in the SJS/TEN, 58.5 ± 2.1 in the DRESS, and 63.0 ± 12.7 in the AGEP groups), while the patients recruited in the control group comprised of 106 men (57.9%) and 77 women (42.1%) with an average age of 42.6 ± 13.6 years.

The mean exposure time to beta-lactam antibiotics until the occurrence of first signs of SJS/TEN (onset or latency period) was 6.1 ± 5.8 (range from 1 to 18) days, whereas those of beta-lactam antibiotics-related DRESS and AGEP were 25.5 ± 23.3 (ranging from 9 to 42) days and 7.3 ± 7.8 (ranging from 2 to 23) days, respectively. The most common comorbidities in SCARs cases included hypertension (25.8%), diabetes (25.8%), chronic renal disease (12.9%), and liver dysfunction (6.5%). For the mucosal involvement in SCARs cases, the mucosal involvements of oral (76.2%) and ocular (52.4%) were mostly reported in SJS/TEN patients. Oral mucosal involvement was found in only one (1/2, 50.0%) patient with DRESS; however, no mucosal involvement was found in AGEP patients.

The mean duration of hospital stay for SCARs treatment was 13.8 ± 15.3 (ranging from 3 to 59) days, in which the longest mean hospital stay was reported in the TEN group among the SJS, SJS/TEN overlap, DRESS, and AGEP groups. The cost of treatment in the TEN group was the highest among the other groups. Only one SCARs patient died in the hospital from sepsis, which may be complication from the SJS/TEN overlap episodes. All data are shown in Table 1.

**TABLE 1:** Demographic and clinical data in beta-lactam antibiotics-related severe cutaneous adverse reactions.

Characteristics	SJS	SJS/TEN overlap	TEN	Total SJS/TEN	AGEP	DRESS	Total SCARs
Demographic data							
*N*	*13*	*1*	*7*	*21*	*8*	*2*	*31*
Age, years							
Mean (SD)	45.4 (27.0)	69	47.7 (21.4)	47.3 (24.5)	63 (12.7)	58.5 (2.1)	52.1 (22.1)
Median [range]	53 [3–82]	NA	49 [8–74]	53 [3–82]	58 [50–82]	58.5 [57–60]	57 [3–82]
Sex, *n* (%)							
Male	5 (38.5)	0 (0)	1 (14.3)	6 (28.6)	2 (25)	1 (50)	9 (29.0)
Female	8 (61.5)	1 (100)	6 (85.7)	15 (71.4)	6 (75)	1 (50)	22 (70.8)
Onset of SCARs, days							
*n* (available data)	*13*	*1*	*6*	*20*	*6*	*2*	*28*
Mean (SD)	4.4 (3.9)	5	9.5 (8.1)	6.1 (5.8)	7.3 (7.8)	25.5 (23.3)	7.7 (9.0)
Median [range]	4 [1–14]	NA	10.5 [1–18]	5 [1–18]	4 [2–23]	25.5 [9–42]	4.5 [1–42]
Mucosal involvement, *n* (%)							
*n* (available data)	*13*	*1*	*7*	*21*	*8*	*2*	*31*
Oral	10 (76.9)^a^	1 (100)	5 (71.4)^a^	16 (76.2)^a^	0 (0)	1 (50)	17 (54.8)
Ocular	6 (46.2)^a^	1 (100)	4 (57.1)^a^	11 (52.4)^a^	0 (0)	0 (0)	11 (35.5)
Genital	4 (30.8)	1 (100)	3 (42.9)	8 (38.1)	0 (0)	0 (0)	8 (25.8)
Concomitant drugs^b^, *n* (%)							
*n* (available data)	*13*	*1*	*7*	*21*	*8*	*2*	*31*
Yes	6 (46.2)	1 (100)	4 (57.1)	11 (52.4)	2 (25)	2 (100)	15 (48.4)
Outcome from SCARs							
Duration of hospital stay for the treatment of SCARs, days							
*n* (available data)	*9*	*1*	*3*	*13*	*4*	*1*	*18*
Mean (SD)	13 (17.8)	17	26 (18.3)	16.31 (17.3)	7.7 (5.9)	5	13.8 (15.3)
Median [range]	7 [3–59]	NA	30 [6–42]	8 [3–59]	6 [3–16]	NA	7.5 [3–59]
Deceased cases, n (%)	0 (0)	1 (100)	0 (0)	1 (4.8)	0 (0)	0 (0)	1 (3.2)
Cost of treatment							
*n* (available data)	5	1	2	8	4	0	12
Mean (SD)	46,263.7 (79,892.7)	79,961	225,726.5 (215,879.0)	95,350.56 (130,073.7)	9,170.5 (9,744.3)	NA	66,623.88 (112,219.1)
Median [range]	12,081 [3,778–188,600]	NA	225,762 [73,113–378,412]	47,486 [3,778–378,412]	7,307.5 [220–21,847]	NA	169,64.3 [220–378,412]

AGEP, acute generalized exanthematous pustulosis; DRESS, drug reactions with eosinophilia and systemic symptoms; NA, not available; SCARs, severe cutaneous adverse drug reactions; SJS, Stevens–Johnson syndrome; SD, standard deviation; TEN, toxic epidermal necrolysis.

^a^ *p* < 0.05; statistically significant when compared with AGEP patients.

^b^ All concomitant drug was evaluated by clinical scoring systems including the Naranjo ADR score for all cases and the ALDEN score for SJS/TEN.

### 3.2 Laboratory data of SCARs patients

Among 31 patients with beta-lactam antibiotics-related SCARs, 15–20% of SCARs patients, particularly patients in the SJS/TEN group, had liver involvement presented with elevated AST or ALT levels (100–500 units/L). Approximately 17% of SCARs patients had kidney involvement. Moreover, hematologic abnormality, neutrophilia, was found in 83% of patients in the AGEP group, while eosinophilia was mainly found in beta-lactam antibiotics-related DRESS patients, followed by AGEP and SJS/TEN patients (Table 2). In addition, fever, enlarged lymph nodes, and atypical lymphocytes were found in two DRESS patients recruited into this study.

**TABLE 2:** Laboratory data of beta-lactam antibiotics-related SCARs patients.

	Phenotypes, number of cases (%)			
Laboratory investigations	SJS/TEN, 21 (67.7)	AGEP, 8 (25.8)	DRESS, 2 (6.5)	Total SCARs, 31 (100.0)
Liver function				
*n* (available data)	*14*	*4*	*2*	*20*
AST (units/L)				
<100	10 (71.4)	4 (100)	2 (100)	16 (80)
100–500	4 (28.6)	0 (0)	0 (0)	4 (20)
501–1,000	0 (0)	0 (0)	0 (0)	0 (0)
ALT (units/L)				
<100	11 (78.6)	4 (100)	2 (100)	17 (85.0)
100–500	3 (21.4)	0 (0)	0 (0)	3 (15.0)
501–1,000	0 (0)	0 (0)	0 (0)	0 (0)
Kidney function				
*n* (available data)	*15*	*6*	*2*	*23*
Acute kidney injury^a^	2 (13.3)	2 (33.3)	0 (0)	4 (17.4)
Hematologic function				
*n* (available data)	*15*	*6*	*2*	*23*
WBC (/µL)				
<11,000/µL	11 (73.3)	2 (33.3)	0 (0)	13 (56.5)
≥11,000/µL	4 (26.7)	4 (66.7)	2 (100)	10 (43.5)
Eosinophils (%)				
<5%	13 (86.7)	4 (66.7)	1 (50)	18 (78.3)
≥5%	2 (13.3)	2 (33.3)	1 (50)	5 (21.7)
Neutrophils (%)				
<80%	10 (66.7)	1 (16.7)	1 (50)	12 (52.2)
≥80%	5 (33.3)	5 (83.3)	1 (50)	11 (47.8)

AGEP, acute generalized exanthematous pustulosis; ALT, alanine aminotransferase; AST, aspartate aminotransferase; DRESS, drug reactions with eosinophilia and systemic symptoms; NA, not available; SCARs severe cutaneous adverse drug reactions; SJS, Stevens–Johnson syndrome; SD, standard deviation; TEN, toxic epidermal necrolysis; WBC, white blood cell.

^a^ Creatinine value was higher than 2.25 mg/dL when SCARs occurred.

### 3.3 Causative beta-lactam antibiotics for SCARs

**FIGURE 1:**
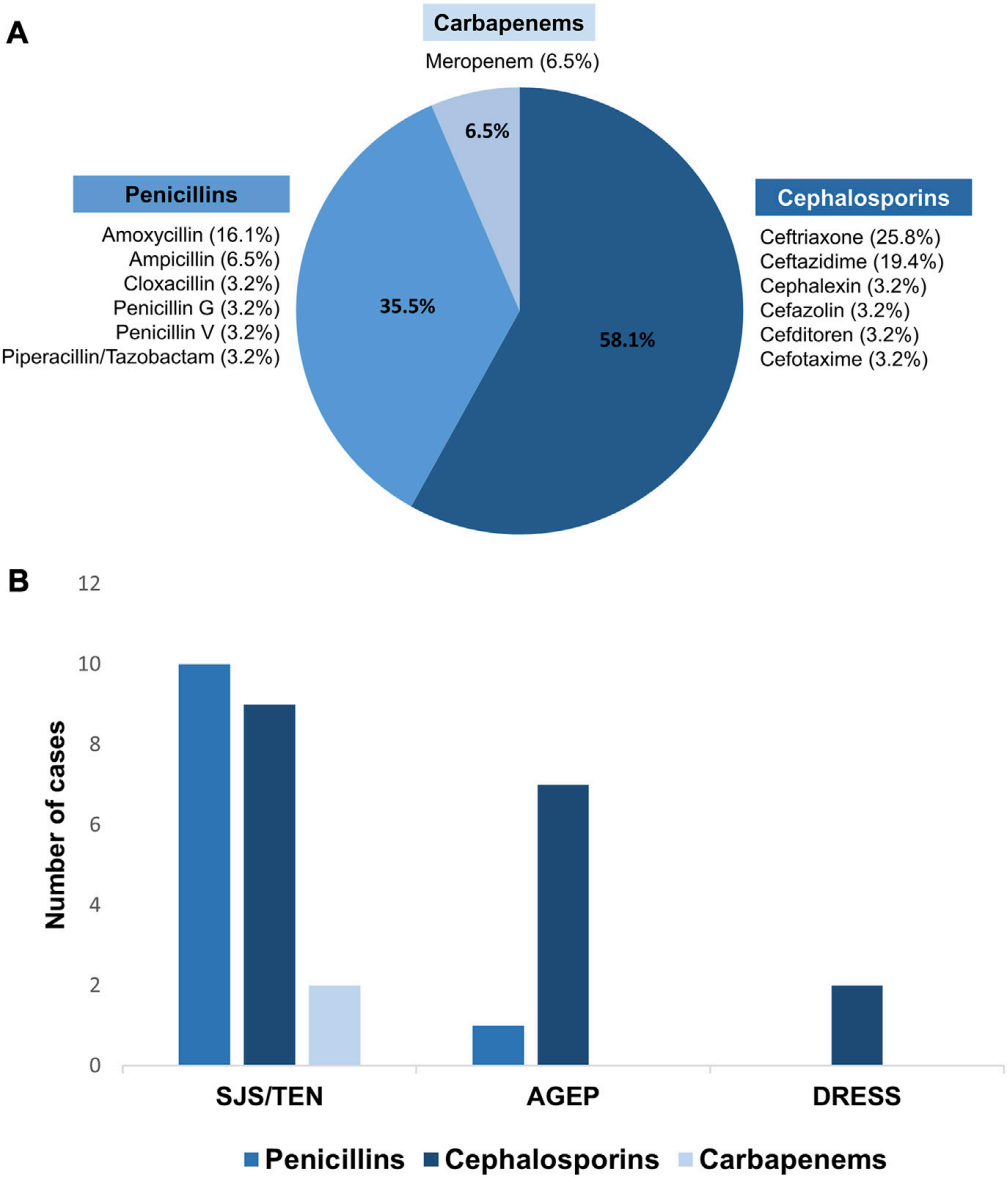
Causative beta-lactam antibiotics for SCARs. **(A)** Percentages of causative beta-lactam antibiotics-related SCARs in the Thai population; **(B)** comparison of the causative beta-lactam antibiotics among SCARs phenotypes.

Three groups of beta-lactam antibiotics were found as causative drugs of SCARs in this present study. Cephalosporins were the most common causative drug of beta-lactam antibiotics-related SCARs, accounting for 58.1% (18/31 patients), followed by penicillins (35.5%, 11/31 patients) and carbapenems (6.5%, 2/31 patients), as shown in Figure 1. Based on the phenotypic profile of SCARs, penicillins were the predominant antibiotic class responsible for SJS/TEN (47.6%, 10/21 patients). Among these cases, the primary causative drug was amoxicillin (5/10 patients), succeeded by ampicillin (2/10 patients). Moreover, cephalosporins constituted the prevailing class of causative beta-lactam antibiotics for AGEP, encompassing 87.5% (7/8 patients), as well as DRESS, comprising 100.0% (2/2 patients). In cases of AGEP, the foremost causative drug was ceftriaxone (4/7 patients), followed by ceftazidime, cefotaxime, and cefazolin. The causative drugs of DRESS were ceftriaxone and ceftazidime.

It is noteworthy that among the cohort of 31 patients, 15 individuals were found to have concomitant drug administration during the manifestation of SCARs (Table 1). The evaluation of all concomitant medications was conducted utilizing the established clinical scoring systems, encompassing the Naranjo ADR score for all cases and ALDEN score for SJS/TEN. Notably, it was observed that solely beta-lactam antibiotics exhibited a score indicative of a probable ADR based on both the Naranjo and ALDEN scores.

### 3.4 Associations between genetic polymorphisms of *HLA* and beta-lactam antibiotics-related SCARs

Genotyping results of *HLA* class I (*HLA-A*, *HLA-B*, and *HLA-C*) and *HLA* class II alleles (*HLA-DRB1*, *HLA-DQA1*, and *HLA-DQB1*) revealed that 34 alleles for *HLA-A*, 53 alleles for *HLA-B*, 36 alleles for *HLA-C*, 29 alleles for *HLA-DRB1*, 10 alleles for *HLA-DQA1*, and 15 alleles for *HLA-DQB1* were identified in this study.

The results from the *HLA* genotyping data of the SCARs group and controls revealed that the carrier frequencies of six *HLA* alleles including HLA-A*01:01, HLA-B*50:01, HLA-C*06:02, HLA-DRB1*15:01, HLA-DQA1*03:01, and HLA-DQB1*03:02, were higher in the SCARs group (*p* < 0.05) (Table 3). Among these six alleles, the highest risk was observed in patients with the HLA-B*50:01 allele (approximately 12.6-fold significantly higher compared with those who did not carry this allele). Moreover, the higher risk of beta-lactam antibiotics-related SCARs was observed in patients who carried the HLA-A*01:01 allele (OR = 4.8, 95% CI = 1.0–22.6, *p* = 0.047), the HLA-C*06:02 allele (OR = 5.7, 95% CI = 1.6–19.9, *p* = 0.011), the HLA-DRB1*15:01 allele (OR = 2.8, 95% CI = 1.1–7.2, *p* = 0.039), the HLA-DQA1*03:01 allele (OR = 4.6, 95% CI = 1.4–14.9, *p* = 0.013), or the HLA-DQB1*03:02 allele (OR = 5.8, 95% CI = 1.5–22.0, *p* = 0.012) (Table 3). However, it should be noted that these associations did not reach a statistically significant difference after Bonferroni’s correction.

**TABLE 3:** List of odds ratios (ORs) and 95% confidence intervals (CIs) in individuals who carried candidate *HLA* class I and II alleles compared with the controls.

*HLA* alleles	Controls (*n* = 183)	SCARs (*n* = 31)			SJS/TEN (*n* = 21)			AGEP (*n* = 8)			DRESS (*n* = 2)		
	Carriers (%)	Carriers (%)	OR [95% CI]	*p*-value	Carriers (%)	OR [95% CI]	*p*-value	Carriers (%)	OR [95% CI]	*p*-value	Carriers (%)	OR [95% CI]	*p*-value
*HLA-A* alleles													
A*11:01	78 (42.6)	8 (25.8)	0.5 [0.2–1.1]	0.112	**3 (14.3)**	**0.2 [0.1–0.8]**	**0.017**	3 (37.5)	0.8 [0.2–3.5]	1.000	2 (100.0)	4.0 [0.4–39.4	0.318
A*01:01	4 (2.2)	**3 (9.7)**	**4.8 [1.0–22.6]**	**0.047**	2 (9.5)	4.7 [0.8–27.4]	0.118	1 (12.5)	6.4 [0.6–64.9]	0.194	0 (0.0)	NA	NA
A*02:06	2 (1.1)	2 (6.5)	6.2 [0.9–46.1]	0.101	**2 (9.5)**	**9.5 [1.3–71.5]**	**0.028**	0 (0.0)	NA	NA	0 (0.0)	NA	NA
A*02:05	0 (0.0)	1 (3.2)	11.9 [1.1–134.9]	0.061	0 (0.0)	NA	NA	**1 (12.5)**	**46.0 [3.8–561.9]**	**0.007**	0 (0.0)	NA	NA
A*02:07	59 (32.2)	**1 (3.2)**	**0.1 [0.0–0.5]**	**3.7 × 10** ^**−4**^ ^a^	**1 (4.8)**	**0.1 [0.0–0.8]**	**0.009**	0 (0.0)	NA	NA	0 (0.0)	NA	NA
A*02:11	1 (0.5)	1 (3.2)	6.1 [0.37–99.62]	0.269	0 (0.0)	NA	NA	**1 (12.5)**	**26.00 [1.5–459.8]**	**0.026**	0 (0.0)	NA	NA
A*03:02	0 (0.0)	1 (3.2)	11.9 [1.1–134.9]	0.061	**1 (4.8)**	**17.5 [1.5–201.6]**	**0.033**	0 (0.0)	NA	NA	0 (0.0)	NA	NA
*HLA-B* alleles													
B*57:01	2 (1.1)	2 (6.5)	6.2 [0.9–46.1]	0.101	**2 (9.5)**	**9.5 [1.3–71.5]**	**0.028**	0 (0.0)	NA	NA	0 (0.0)	NA	NA
B*37:01	0 (0.0)	1 (3.2)	11.9 [1.1–134.9]	0.061	0 (0.0)	NA	NA	**1 (12.5)**	**46.0 [3.8–561.9]**	**0.007**	0 (0.0)	NA	NA
B*38:01	0 (0.0)	1 (3.2)	11.9 [1.1–134.9]	0.061	0 (0.0)	NA	NA	**1 (12.5)**	**46.0 [3.8–561.9]**	**0.007**	0 (0.0)	NA	NA
B*46:02	0 (0.0)	1 (3.2)	11.9 [1.1–134.9]	0.061	**1 (4.8)**	**17.5 [1.5–201.6]**	**0.033**	0 (0.0)	NA	NA	0 (0.0)	NA	NA
B*50:01	1 (0.5)	**2 (6.5)**	**12.6 [1.1–142.9]**	**0.042**	0 (0.0)	NA	NA	**2 (25.0)**	**60.7 [4.8–765.0]**	**0.005**	0 (0.0)	NA	NA
*HLA-C* alleles													
C*06:02	6 (3.3)	**5 (16.1)**	**5.7 [1.6–19.9]**	**0.011**	**3 (14.3)**	**4.9 [1.1–21.4]**	**0.034**	**2 (25.0)**	**9.8 [1.6–59.2]**	**0.038**	0 (0.0)	NA	NA
C*04:06	3 (1.6)	2 (6.5)	4.1 [0.7–25.8]	0.154	1 (4.8)	3.0 [0.3–30.2]	0.355	0 (0.0)	NA	NA	**1 (50.0)**	**60.0 [3.0–1,202.1]**	**0.043**
C*03:09	0 (0.0)	1 (3.2)	11.9 [1.1–134.9]	0.061	0 (0.0)	NA	NA	**1 (12.5)**	**46.0 [3.8–561.9]**	**0.007**	0 (0.0)	NA	NA
*HLA-DRB1* alleles													
DRB1*15:01	20 (10.9)	**8 (25.8)**	**2.8 [1.1–7.2]**	**0.039**	5 (23.8)	2.6 [0.8–7.7]	0.149	**3 (37.5)**	**4.9 [1.1–22.0]**	**0.039**	0 (0.0)	NA	NA
DRB1*04:05	20 (10.9)	6 (19.4)	2.0 [0.7–5.3]	0.230	3 (14.3)	1.4 [0.4–5.0]	0.713	1 (12.5)	1.2 [0.1–10.0]	1.000	**2 (100.0)**	**23.4 [2.3–235.6]**	**0.007**
DRB1*11:01	9 (4.9)	1 (3.2)	0.6 [0.1–5.3]	1.000	0 (0.0)	NA	NA	0 (0.0)	NA	NA	**1 (50.0)**	**19.3 [1.1–334.8]**	**0.042**
*HLA-DQA1* alleles													
DQA1*03:01	6 (6.0)^b^	**7 (22.6)**	**4.6 [1.4–14.9]**	**0.013**	4 (19.1)	3.7 [0.9–14.5]	0.070	2 (25.0)	5.2 [0.9–31.6]	0.108	1 (50.0)	15.7 [0.9–282.5]	0.133
*HLA-DQB1* alleles													
DQB1*03:02	4 (4.0)^b^	**6 (19.4)**	**5.8 [1.5–22.0]**	**0.012**	**5 (23.8)**	**7.5 [1.8–30.9]**	**0.008**	1 (12.5)	3.4 [0.3–35.0]	0.325	0 (0)	NA	NA
DQB1*04:01	11 (11.0)^b^	3 (9.7)	0.9 [0.2–3.3]	1.000	0 (0.0)	NA	NA	1 (12.5)	1.2 [0.1–10.3]	1.000	**2 (100.0)**	**22.5 [2.2–234.0]**	**0.009**

AGEP, acute generalized exanthematous pustulosis; DRESS, drug reactions with eosinophilia and systemic symptoms; NA, not available; SCARs, severe cutaneous adverse drug reactions; SJS, Stevens–Johnson syndrome; SD, standard deviation; TEN, toxic epidermal necrolysis.

*p*-values were calculated using Fisher’s exact test. *p*-value < 0.05 was highlighted in bold.

The corrected *p*-values (Pc) for the multiple comparisons of all alleles were calculated using Bonferroni’s correction (34 for *HLA-A*, 53 for *HLA-B*, 36 for *HLA-C*, 29 for *HLA-DRB1*, 10 for *HLA-DQA1*, and 15 for *HLA-DQB1*).

^a^ Statistical significance was found after Bonferroni’s correction.

^b^ Data obtained from the published information by Satapornpong et al. (2020) (*n* = 100).

In contrast, the risk of beta-lactam antibiotics-related SCARs was statistically significantly lower in patients who carried the HLA-A*02:07 allele (OR = 0.1, 95% CI = 0.0–0.5, *p* = 3.7 × 10^−4^, Pc = 0.012).

In the subgroup analysis, the frequencies of patients who carried *HLA* alleles, including HLA-A*02:06, HLA-A*03:02, HLA-B*57:01, HLA-B*46:02, HLA-C*06:02, and HLA-DQB1*03:02, were significantly higher in the SJS/TEN group compared with the control group (*p* < 0.05) (Table 3). Moreover, the risk of SJS/TEN was approximately 4.9- to 17.5-fold higher in patients who carried one of these six alleles compared with the controls (HLA-A*02:06 allele, OR = 9.5, 95% CI = 1.3–71.5, *p* = 0.028; HLA-A*03:02 allele, OR = 17.5, 95% CI = 1.5–201.6, *p* = 0.033; HLA-B*57:01 allele, OR = 9.5, 95% CI = 1.3–71.5, *p* = 0.028; HLA-B*46:02 allele, OR = 17.5, 95% CI = 1.5–201.6, *p* = 0.033; HLA-C*06:02 allele, OR = 4.9, 95% CI = 1.1–21.4, *p* = 0.034; and HLA-DQB1*03:02 allele, OR = 7.5, 95% CI = 1.8–30.9, *p* = 0.008) (Table 3). Interestingly, the risk of SJS/TEN related to beta-lactam antibiotics was lower in patients who carried either the HLA-A*11:01 (OR = 0.2, 95% CI = 0.1–0.8, *p* = 0.017) or HLA-A*02:07 allele (OR = 0.1, 95% CI = 0.0–0.8, *p* = 0.009). However, it should be noted that these associations did not reach a statistically significant difference after Bonferroni’s correction.

For the AGEP group, the carrier frequencies of eight *HLA* alleles, namely, HLA-A*02:05, HLA-A*02:11, HLA-B*37:01, HLA-B*38:01, HLA-B*50:01, HLA-C*06:02, HLA-C*03:09, and HLA-DRB1*15:01, were significantly higher compared with the control group (*p* < 0.05) (Table 3). Among these eight alleles, the HLA-B*50:01 allele showed the highest risk of beta-lactam antibiotics-related AGEP*.* The strength of associations is shown in Table 3. The frequency of patients who carried the HLA-B*50:01 allele in the AGEP group was approximately 2.3-fold higher (2/8, 25.0%) compared with that in the control group (1/183, 0.5%), and the risk of AGEP for patients who carried the HLA-B*50:01 allele was 60.7 (95% CI = 4.8–765.0, *p* = 0.005). However, this did not reach a statistically significant difference after Bonferroni’s correction.

When considering the associations between *HLA* and beta-lactam antibiotics-related DRESS, it was found that four *HLA* alleles including HLA-C*04:06, HLA-DRB1*04:05, HLA-DRB1*11:01, and HLA-DQB1*04:01, showed the significantly higher frequencies in the DRESS group compared with the control group (*p* < 0.05) (Table 3). Among these four alleles, the HLA-C*04:06 allele showed the highest risk of DRESS with an OR of 60.0 (95% CI = 3.0–1202.1, *p* = 0.043). Moreover, the higher risk of DRESS was observed in patients who carried the HLA-DRB1*04:05 allele (OR = 23.4, 95% CI = 2.3–235.6, *p* = 0.007), the HLA-DRB1*11:01 allele (OR = 19.3, 95% CI = 1.1–334.8, *p* = 0.042), or the HLA-DQB1*04:01 allele (OR = 22.5, 95% CI = 2.2–234.0, *p* = 0.009) (Table 3). However, it should be noted that these associations did not reach a statistically significant difference after Bonferroni’s correction.

## 4 Discussion

The majority of SCARs cases identified in the study population were SJS/TEN patients, followed by AGEP and DRESS patients. The most common beta-lactam antibiotics related to SCARs were cephalosporins, followed by penicillins and carbapenems. These findings were inconsistent with a previous study in Taiwan, which reported penicillins as the most common cause of SCARs (Lin et al., 2014). Furthermore, a previous study in Australia indicated that penicillins were the most common cause of cutaneous adverse drug reactions, including SCARs phenotypes (Trubiano et al., 2016). When considering the phenotype of SCARs, penicillins were the most frequent causative antibiotic class for SJS/TEN patients, whereas cephalosporins were the most common drug related to DRESS and AGEP.

The results of the clinical characteristics of SCARs patients in this study demonstrated that most of the SCARs patients were female, and the finding was consistent with the previous studies that the frequency of documented drug allergy is higher in women in drug allergies such as antibiotic allergies in adults (Blumenthal et al., 2019) and allopurinol-induced SCARs (Saksit et al., 2017b). The mean exposure time to beta-lactam antibiotics and the first signs of SCARs (latency) varies depending on SCARs phenotypes. DRESS showed the longest latency among other phenotypes, which was consistent with the previous studies that reported about DRESS caused by co-trimoxazole (Nakkam et al., 2022b), allopurinol (Saksit et al., 2017b), or phenytoin (Tassaneeyakul et al., 2016). In addition, patients with beta-lactam antibiotics-related TEN showed the longest duration of hospital stay for the treatment of SCARs and the highest cost of treatment among other SCARs phenotypes. The mucosal involvements and systemic manifestations including hepatitis and renal insufficiency were mainly noted in the SJS/TEN patients. In contrast, the hematological abnormalities including eosinophilia and neutrophilia were noted in the DRESS and AGEP patients, respectively. Of these 31 cases, only one SJS/TEN overlap patient died in the hospital due to sepsis that may be a complication from the SJS/TEN overlap episodes. This patient was a 69-year-old Thai female patient, prescribed with ceftriaxone for the treatment of infective diarrhea, and norfloxacin and metronidazole were prescribed as concurrent medications, in which only ceftriaxone showed the score of probable ADR based on the Naranjo score and probable SJS/TEN based on the ALDEN score. The SJS/TEN overlap was developed after ceftriaxone exposure for 5 days, and the duration of hospital stay of this patient was 17 days. Interestingly, this patient carried the HLA-B*57:01 and HLA-C*06:02 alleles in which these two alleles were proposed as candidate genetic markers for beta-lactam antibiotics-related SJS/TEN in this present study.

To the best of our knowledge, this association study is the first and largest well-defined case–control study of genetic polymorphisms of *HLA* class I and II and beta-lactam antibiotics-related SCARs. Of the *HLA* genetic polymorphisms determined, six *HLA* alleles including HLA-A*01:01, HLA-B*50:01, HLA-C*06:02, HLA-DRB1*15:01, HLA-DQA1*03:01, and HLA-DQB1*03:02, were associated with the increased risk of beta-lactam antibiotics-related SCARs. These results were not in line with the previous study in Thai children, in which HLA-C*04:06, HLA-C*08:01, and HLA-DRB1*04:06 were significantly associated with beta-lactam antibiotics-induced delayed hypersensitivity (Singvijarn et al., 2019). It is important to note that the previous study included patients with various phenotypes of delayed hypersensitivity, such as urticaria, maculopapular eruption, fixed drug eruption, SJS, and DRESS (Singvijarn et al., 2019). In the study, there was only one ampicillin/sulbactam-induced SJS patient who carried HLA-A*11:01/24:02, HLA-B*18:01/52:01, HLA-C*05:01/12:02, and HLA-DRB1*04:03/04:84 alleles and one meropenem-induced DRESS patient who carried HLA-A*24:02/74:01, HLA-B*15:02/15:02, HLA-C*08:01/08:01, and HLA-DRB1*12:02/14:22 alleles (Singvijarn et al., 2019).

Regarding several *HLA* risk alleles of beta-lactam antibiotics related to SCARs identified in this present study, the linkage disequilibrium (LD) analysis of the study population was also performed. The results showed that there was no significance in the linkage disequilibrium (LD) of any two, three, four, or five alleles of these six *HLA* risk alleles (HLA-A*01:01, HLA-B*50:01, HLA-C*06:02, HLA-DRB1*15:01, HLA-DQA1*03:01, and HLA-DQB1*03:02) (D’ < 1 and *r* ^2^ < 1) (data not shown), suggesting that these identified *HLA* risk alleles were not linkage disequilibrium.

It is well recognized that the immunopathogenesis and clinical characteristics of each phenotype of SCARs are markedly different (Gibson et al., 2023). Some previous studies have proposed specific associations between *HLA* alleles and drug-induced SCARs for certain phenotypes, such as HLA-B*15:02 for SJS/TEN induced by carbamazepine (Hung et al., 2006; Nakkam et al., 2022a) and HLA-B*13:01 for DRESS induced by co-trimoxazole (Sukasem et al., 2020; Wang et al., 2021; Nakkam et al., 2022b). Therefore, subgroup analysis based on SCARs phenotypes was performed in the present study, and the results showed that six *HLA* alleles including HLA-A*02:06, HLA-A*03:02, HLA-B*57:01, HLA-B*46:02, HLA-C*06:02, and HLA-DQB1*03:02, were associated with SJS/TEN. However, eight *HLA* alleles including HLA-A*02:05, HLA-A*02:11, HLA-B*37:01, HLA-B*38:01, HLA-B*50:01, HLA-C*06:02, HLA-C*03:09, and HLA-DRB1*15:01, were associated with AGEP. Moreover, four *HLA* alleles including HLA-C*04:06, HLA-DRB1*04:05, HLA-DRB1*11:01, and HLA-DQB1*04:01 were associated with DRESS related to beta-lactam antibiotics.

Among six *HLA* alleles associated with SJS/TEN, the HLA-DQB1*03:02 allele showed the highest prevalent in SJS/TEN cases (5/21, 23.8%) compared with other alleles, and the higher risk of SJS/TEN was observed in patients who carried this allele (OR = 7.5, 95% CI = 1.8–30.9, *p* = 0.008). Interestingly, when using the *HLA* data from a larger cohort of a general Thai population as a control group (Satapornpong et al., 2020), the association between the HLA-DQB1*03:02 allele and SJS/TEN group was still noticed, with an OR of 3.7 (95% CI = 1.3–10.5, *p* = 0.026). This finding suggested that the HLA-DQB1*03:02 allele may play some role in the development of SJS/TEN related to beta-lactam antibiotics.

Previous studies reported that the HLA-B*57:01 allele was statistically significantly associated with flucloxacillin-induced liver injury (Daly et al., 2009; Puig et al., 2020). Flucloxacillin is one of the beta-lactam antibiotics that has been associated with severe immune-mediated drug-induced liver injury caused by an influx of T lymphocytes targeting liver cells potentially recognizing drug-haptenated peptides in the context of HLA-B*57:01 (Puig et al., 2020). Moreover, the HLA-B*57:01 allele was reported as a valid genetic marker for abacavir hypersensitivity which is one of T-cell-mediated adverse drug reactions (Mallal et al., 2002; Mallal et al., 2008). Consistent with those observed in these previous studies (Mallal et al., 2002; Mallal et al., 2008; Daly et al., 2009; Puig et al., 2020), this present study found that HLA-B*57:01 was identified as the risk allele for SJS/TEN related to beta-lactam antibiotics (OR = 9.5, 95% CI = 1.3–71.5, *p* = 0.028). According to the immunopathogenesis of these immune-mediated adverse drug reactions, this finding suggested that HLA-B*57:01 may play some roles in the development of T-cell-mediated adverse reactions induced by beta-lactam antibiotics.

The previous study in a Japanese population reported that HLA-A*02:06 was strongly associated with cold medicine-related SJS with severe ocular complications, with an OR of 5.7 (*p* = 2.8 × 10^−16^, Pc = 4.8 × 10^−15^) (Ueta et al., 2014), and the result was further confirmed with another Japanese cohort using next-generation sequencing (NGS)-based HLA typing (Nakatani et al., 2019). Interestingly, this present study found the association between HLA-A*02:06 and beta-lactam antibiotic-induced SJS/TEN, with an OR of 9.5 (95% CI = 1.3–71.5, *p* = 0.028). The association between HLA-A*02:06 and beta-lactam antibiotics-related SCARs deserves further investigation.

The highest risk of AGEP induced by beta-lactam antibiotics was found in patients who carried the HLA-B*50:01 allele compared with the controls who did not carry this allele. Moreover, the risk of AGEP was determined using *HLA* data from a larger cohort of a general Thai population as the control group (Satapornpong et al., 2020), and the results remained the same for the HLA-B*50:01 allele in which the risk of AGEP was statistically significantly higher in the patients who carried the HLA-B*50:01 allele, with an OR of 201.9 (95% CI = 18.6–2,187.7, *p* = 2.6 × 10^−5^, Pc = 1.4 × 10^−3^). It should be noted that this allele was uncommon in a Thai population, and the allele frequency was approximately 0–0.28% (Nakkam et al., 2018; Satapornpong et al., 2020). This finding indicated that the HLA-B*50:01 allele may play important roles in the development of AGEP related to beta-lactam antibiotics. However, this association deserves further exploration in larger sample size and other ethnicity.

Interestingly, the results from this study demonstrated that the HLA-C*06:02 allele was apparently associated with both SJS/TEN and AGEP. When combining all phenotypes of SCARs, the HLA-C*06:02 allele was also observed to be the risk allele for SCARs related to beta-lactam antibiotics, with an OR of 5.7 (95% CI = 1.6–19.9, *p* = 0.011) (Table 3).

The risk of DRESS was observed in patients who carried HLA-C*04:06, HLA-DRB1*04:05, HLA-DRB1*11:01, or HLA-DQB1*04:01. Moreover, the risk of beta-lactam antibiotics-related DRESS was significantly higher than that in patients who carried these alleles even when determined using the general Thai population (Satapornpong et al., 2020) as the control group (HLA-C*04:06, OR = 41.7, 95% CI = 2.4–711.0, *p* = 0.010; HLA-DRB1*04:05, OR = 27.8, 95% CI = 2.8–272.6, *p* = 0.004; HLA-DRB1*11:01, OR = 30.3, 95% CI = 1.8–508.4, *p* = 0.018; and HLA-DQB1*04:01, OR = 36.3, 95% CI = 3.7–358.2, *p* = 0.002, Pc = 0.029). It should be noted that only two patients with DRESS were enrolled into this study.

Apart from the *HLA* risk alleles of SCARs related to beta-lactam antibiotics, the results from this study found that the HLA-A*11:01 and HLA-A*02:07 alleles were more common in the controls and the general Thai population (Nakkam et al., 2018; Satapornpong et al., 2020) than in the cases, suggesting that they may protect against beta-lactam antibiotics-related SJS/TEN in this study. Consistent with the previous study in Iranian pediatric patients, HLA-A*11:01 appeared to have protective effect against drug-related-SJS/TEN with an OR of 0.1 (95% CI = 0.008–0.493, *p* = 0.001), and the most common causative drug in this previous study was beta-lactam antibiotics (Esmaeilzadeh et al., 2019). In addition, it was observed that the HLA-A*02:07 allele may protect against the development of SJS/TEN induced by co-trimoxazole in the Thai population (Sukasem et al., 2020; Nakkam et al., 2022b).

It should be noted that there are limitations to the present study: 1) a relatively small sample size of the SCARs cases due to the rarity of beta-lactam antibiotic-related SCARs; 2) the identification of causative drugs in SCARs cases relied on clinical scoring systems (the Naranjo ADR score for all cases and the ALDEN score for SJS/TEN); and 3) *in vitro* or *in vivo* testing was not performed to validate whether beta-lactam antibiotics were indeed the causative agents.

In conclusion, the present study provided data on the clinical characteristics of beta-lactam antibiotics-related SCARs and demonstrated the candidate *HLA* alleles that showed significant associations with specific phenotypes of these drug-related SCARs in a Thai population. The identified *HLA* alleles may potentially serve as valid markers for SCARs related to beta-lactam antibiotics. However, these associations warrant further exploration in larger sample sizes and among other ethnicities.

## Data Availability

The original contributions presented in the study are included in the article/Supplementary Material. Further inquiries can be directed to the corresponding author.
